# Carbon dioxide hydrogenation to aromatic hydrocarbons by using an iron/iron oxide nanocatalyst

**DOI:** 10.3762/bjnano.5.88

**Published:** 2014-06-02

**Authors:** Hongwang Wang, Jim Hodgson, Tej B Shrestha, Prem S Thapa, David Moore, Xiaorong Wu, Myles Ikenberry, Deryl L Troyer, Donghai Wang, Keith L Hohn, Stefan H Bossmann

**Affiliations:** 1 Kansas State University, Department of Chemistry, 201CBC Building, Manhattan, KS 66506, USA, 001-785-532-6817; 2 Kansas State University, Department of Anatomy & Physiology, 130 Coles Hall, Manhattan, KS 66506, USA; 3 University of Kansas, Microscopy and Analytical Imaging Laboratory, 1043 Haworth, Lawrence, KS 66045, USA; 4 Kansas State University, Department of Biological and Agricultural Engineering, 150 Seaton Hall, Manhattan, KS 66506, USA; 5 Kansas State University, Department of Chemical Engineering, 1016 Durland Hall, Manhattan, KS 66506, USA

**Keywords:** aromatic hydrocarbons, carbon dioxide reduction, heterogenous catalysis, iron/iron oxide nanocatalyst

## Abstract

The quest for renewable and cleaner energy sources to meet the rapid population and economic growth is more urgent than ever before. Being the most abundant carbon source in the atmosphere of Earth, CO_2_ can be used as an inexpensive C1 building block in the synthesis of aromatic fuels for internal combustion engines. We designed a process capable of synthesizing benzene, toluene, xylenes and mesitylene from CO_2_ and H_2_ at modest temperatures (*T* = 380 to 540 °C) employing Fe/Fe_3_O_4_ nanoparticles as catalyst. The synthesis of the catalyst and the mechanism of CO_2_-hydrogenation will be discussed, as well as further applications of Fe/Fe_3_O_4_ nanoparticles in catalysis.

## Introduction

The diminishing fossil reserves and the ever-increasing CO_2_ emissions have been of great concern amongst the scientific community. Since the industrial revolution, a significant increase of CO_2_ concentration in the atmosphere because of the combustion of carbon-rich fossil fuels has been witnessed, which consequently leads to global warming and drastic climate changes [1–3]. As a result, the quest for renewable and cleaner energy sources to meet the rapid population and economic growth is more urgent than ever before. Being the most abundant carbon source in the atmosphere of Earth, CO_2_ can be used as a cheap and non-toxic C1 building block in many chemical processes [4–7]. To achieve this goal, CO_2_ should come from the atmosphere and H_2_ should be created by using solar energy from water [8–10]. Iron-based heterogeneous catalysts have been intensively studied for CO_2_ hydrogenation reactions. Earlier research showed that bulk iron and iron oxides catalyze CO_2_ hydrogenation, producing mainly methane. These catalysts were rapidly deactivated due to carbon deposition [11–12]. Doping with promoters such as potassium [13–18], manganese [19–21] and copper [22] had significant effect on both the reactivity and selectivity of the iron-based catalysts. Higher olefins and aliphatic hydrocarbons, as well as improved CO_2_ conversion, were achieved. Al_2_O_3_ was found to be an excellent structural promoter to sustain the catalyst activity of iron-based catalysts by preventing sintering of active particles during the reaction [23–24]. When using zeolites as solid supports, the product distribution was highly dependent on the structure and acidity of the zeolites [25–27]. Iron–zeolite composites were reported as dual functional catalysts, which promoted multistep CO_2_ hydrogenation reactions [28].

In spite of all the efforts to date, the direct formation of aromatic hydrocarbons in a one-step reaction from carbon dioxide, without forming aliphatic hydrocarbons first, remained elusive. Here, we report the selective formation of aromatic hydrocarbons from CO_2_ and H_2_ via a novel iron nanocatalyst.

## Results and Discussion

### Advantages of nanomaterials as heterogeneous catalysts

Due to their unique properties, such as a large surface-area-to-volume ratio, nanomaterials have attracted massive attention in catalysis applications [29–31]. Using newly developed in-situ characterization technologies, detailed atomic- and molecular-level information of the catalytic reaction mechanisms has been revealed [32–33]. Elegant protocols to synthesize monodispersed composite iron-based or iron-containing nanomaterials with controlled size and shape have been developed [34–41]. The application of such materials in cancer diagnosis and cancer treatment, such as MRI and magnetic hyperthermia are intensively studied [42–44]. The use of iron-containing nanomaterials as catalysts for the methanol oxidation reaction [45], and oxygen reduction reaction [46–48] have been reported.

#### Synthesis of Fe/Fe_3_O_4_ nanoparticles

Here we report the selective formation of aromatic hydrocarbons from CO_2_ hydrogenation reactions catalyzed by an Fe/Fe_3_O_4_ nanocatalyst. Recently, Sun’s group reported a facile method for synthesizing highly crystalline Fe/Fe_3_O_4_ nanoparticles [49]. These nanoparticles were found to be robust against deep oxidation because of the formation of a protective crystalline Fe_3_O_4_ shell upon the direct oxidation of the bcc-Fe core. The synthesis of the Fe/Fe_3_O_4_ nanoparticles was slightly modified and scaled up by a factor of three (see Experimental section). To avoid violent Fe(CO)_5_ reflux, three portions of 0.70 mL Fe(CO)_5_ were added to the mixture of the ligands oleylamine and hexadecylammonium hydrochloride (HDA·HCl) and solvent (1-octadecene, ODE) every 20 min at 180 °C instead of adding the iron precursor all at one time. After the third addition, the reaction mixture was kept for 40 min at 180 °C to permit a controlled nanoparticle growth, and then allowed to cool down to rt. After decanting of the supernatant, the nanoparticles that were accumulated on the stirring bar were thoroughly washed with hexane and then ethanol (sonication) to remove the free ligands. The NPs were dried in high vacuum, and the yield based on iron was found to be 95%.

#### CO_2_ hydrogenation reaction

**Figure 1:**
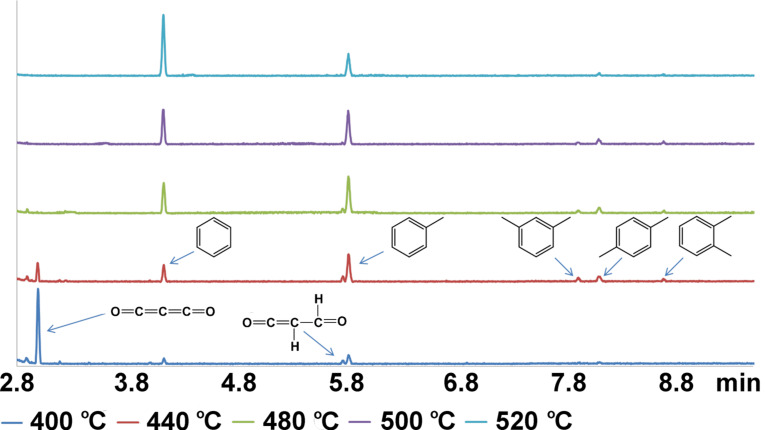
GC profile of the products formed during CO_2_ hydrogenation at different temperatures.

The Fe/Fe_3_O_4_ nanoparticle-catalyzed CO_2_ hydrogenation reaction was performed in a custom-built reactor (see details in the Experimental section). 50 mg Fe/Fe_3_O_4_ nanoparticles were subjected to a continuous supply of (1:1 mol/mol) CO_2_/H_2_ atmosphere at 1 atm pressure. Gas samples were withdrawn from the reactor and analyzed by GC–MS during the reaction. The GC profile of the products during the reaction is shown in Figure 1. Upon heating from rt to 400 °C, traces of butane (2.881 min), benzene (4.135 min) and toluene (5.819 min) were observed, together with a major peak (2.989 min) and minor peak (5.765 min) in the GC profile corresponding to *m*/*z* 68 and 71. We have assigned these two species to carbon suboxide (C_3_O_2_) and its partially reduced form (C_3_O_2_H_3_). Upon raising the temperature to 440 °C, three new peaks appeared at 7.915 min, 8.100 min, 8.688 min, all of these correspond to *m*/*z* 106. By comparing with commercially available standard samples, these three peaks were identified as *m*-xylene, *p*-xylene and *o*-xylene, respectively. The reaction temperature was raised at a rate of 1 °C/min afterwards, and gas samples were subjected to GC–MS analysis every 20 min. We found that, with the increase of reaction temperature, the intensity of the peak at 2.989 min (C_3_O_2_), decreased gradually. At the same time, the intensity of the benzene peak (4.135 min) and toluene peak (5.819 min) increased. At 480 °C, the peak at 2.989 min (C_3_O_2_) disappeared completely, while the peak at 5.765 min (C_3_O_2_H_3_) still persisted until the temperature reached 500 °C. Further increasing temperature to 520 °C led to the decrease of the xylene peaks. The decrease of the toluene peak and the increase of the benzene peak were observed while keeping the reaction at 520 °C for 40 min. A heterogeneous rate constant of 0.00113 s^−1^·g^−1^ for the consumption of CO_2_ at 400 °C was calculated from the kinetic data shown in the Supporting Information File 1 (Figure S1). The catalyst was reused 10 times. No decrease of the catalytic activity was observed. This observation is based on the consumption efficiency of CO_2_ from the gas phase and product analysis by GC–MS.

#### Characterization of the catalysts (TEM, HRTEM, XRD, XPS)

**Figure 2:**
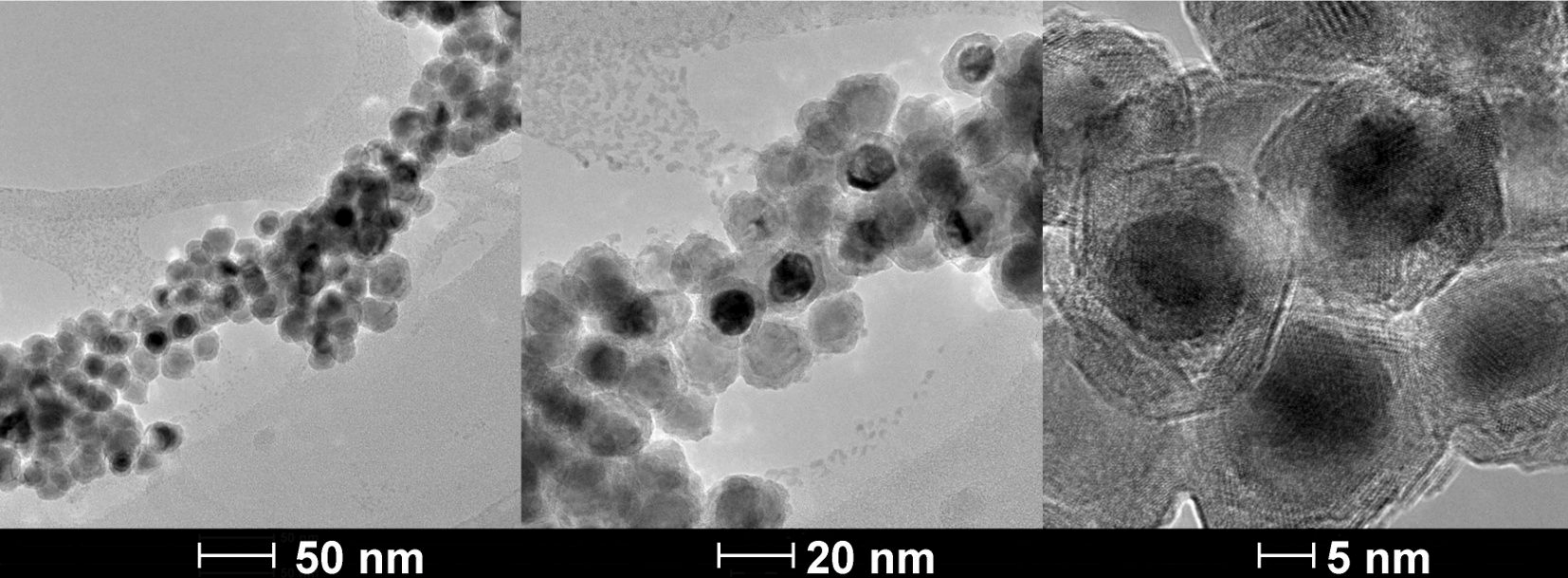
TEM and HRTEM of Fe/Fe_3_O_4_ nanoparticles prepared by thermal decomposition of Fe(CO)_5_ in the presence of oleylamine and HDA·HCl.

The TEM image reveals that the newly synthesized Fe/Fe_3_O_4_ nanoparticles are roughly spherical with a core/shell structure (Figure 2). The mean core diameter is 12 nm, and the shell thickness is 2 nm. HRTEM indicate that each Fe/Fe_3_O_4_ nanoparticle assumes polycrystalline structure with rigid edges.

**Figure 3:**
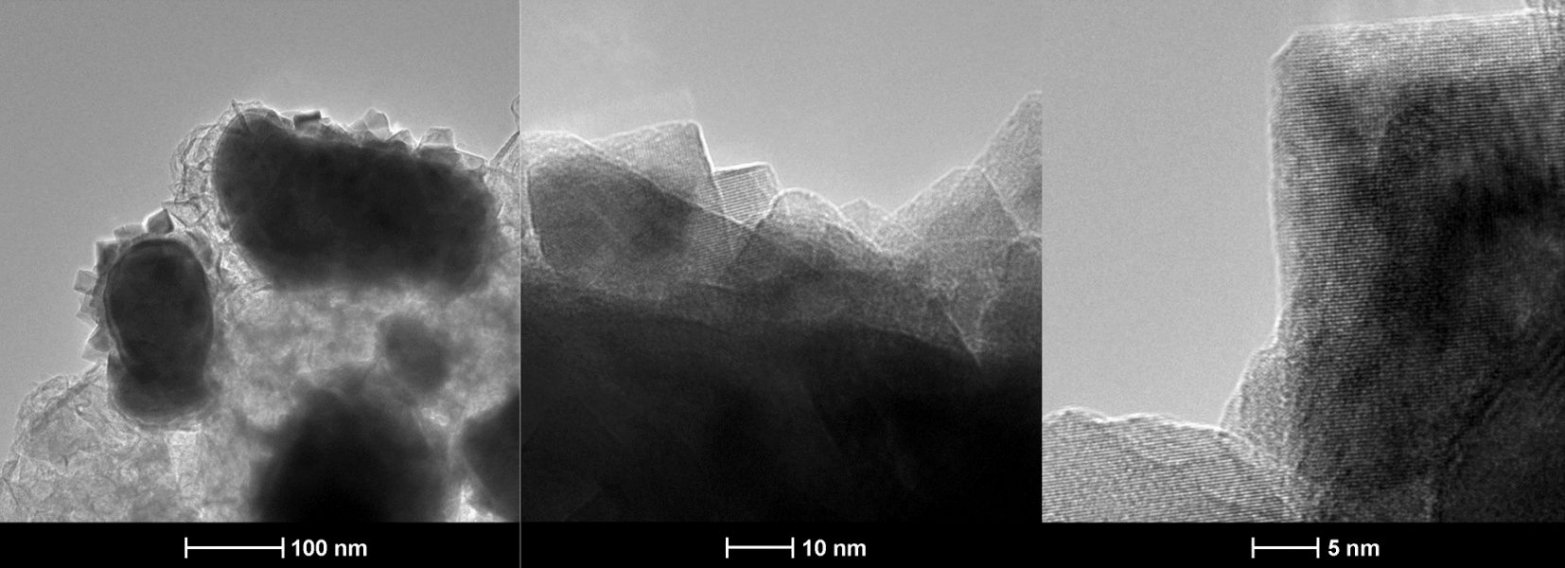
TEM and HRTEM of Fe/Fe_3_O_4_ nanoparticles prepared by thermal decomposition of Fe(CO)_5_ in the presence of oleylamine and HDA·HCl after performing 10 catalytic cycles.

TEM images (Figure 3) of recycled catalyst after 10 runs of reactions shows that the nanoparticles fused to larger irregularly shaped particles with crystalline substructures on the surface. HRTEM reveal that the substructure is polycrystalline.

**Figure 4:**
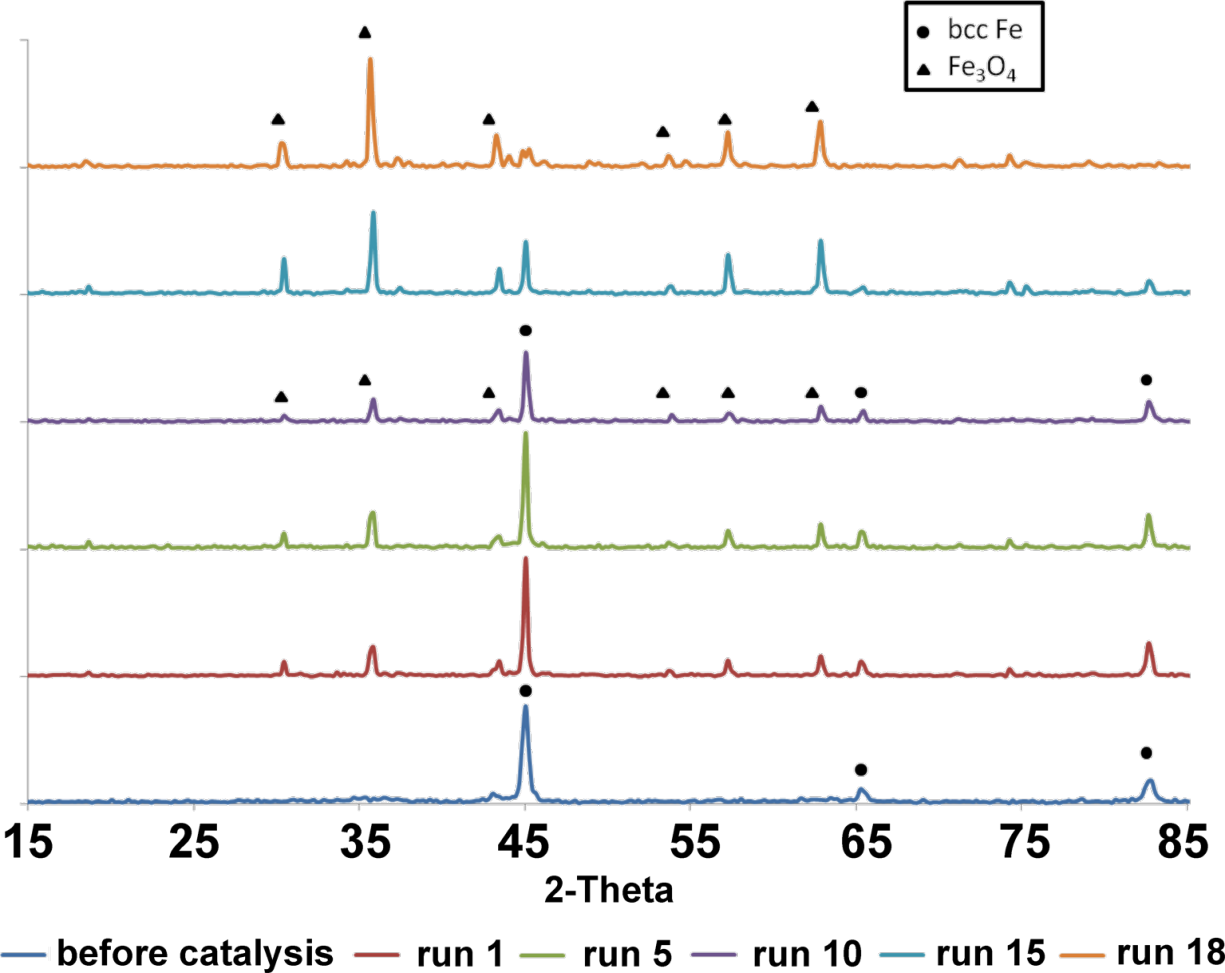
XRD patterns of the Fe/Fe_3_O_4_ nanoparticles as a function of catalytic run (2 h at 400 °C).

The XRD patterns of the Fe/Fe_3_O_4_ nanoparticles as a function of the number of catalytic runs is shown in Figure 4. The XRD characterization of the freshly prepared Fe/Fe_3_O_4_ nanoparticles confirmed the crystalline structure as previously reported [50]: Only the (110) and (200) peaks corresponding to bcc-Fe are observed. XRD characterization of the recycled nanoparticles after each run of catalysis demonstrated that the crystallinity of the catalyst changed from bcc-Fe to a mixture of bcc-Fe and Fe_3_O_4_ (run 1 to run 17), and eventually to Fe_3_O_4_ (after run 18 of catalysis reaction). The observed conversion of bcc-Fe to Fe_3_O_4_ in the presence of H_2_O at the reported temperature is in agreement with literature findings [51].

#### XPS analysis of the surface of the catalyst

**Figure 5:**
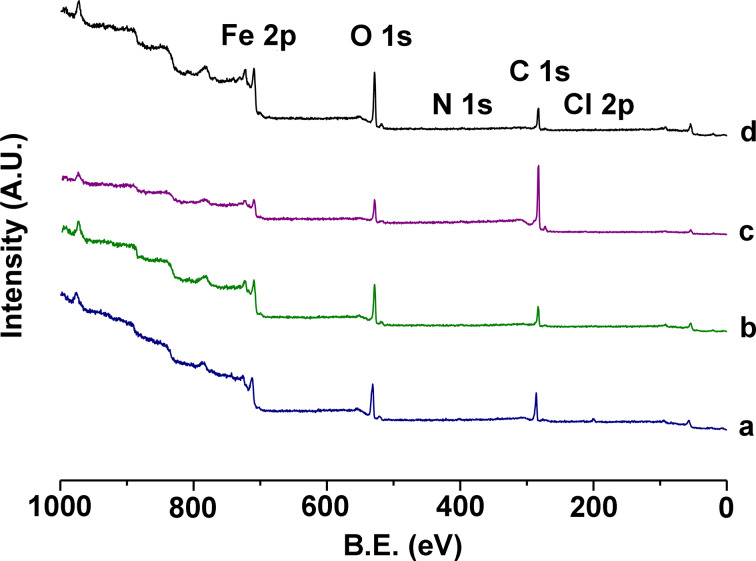
XPS surveys of the catalyst: a) as prepared with HDA synthesis, b) after 5 runs, c) after 10 runs, d) after 6 runs, followed by H_2_ reduction. Catalytic runs were performed for 2 h at 400 °C.

The XPS analysis [52] of the fresh catalyst (Table 1) is consistent with iron oxide that is covered by a molecular layer of oleylamine/HDA·HCl. XPS indicates a decreased carbon composition after the first five runs, followed by an increased carbon loading from run 5 through run 10, as shown in Figure 5. This is consistent with the hypothesis that the surface loses the coordinating HDA ligands during the initial runs. The concurrent loss of nitrogen and chlorine is consistent with this mechanistic assumption. The lost carbon by displacement of HDA is overcompensated during runs 6–10 by the deposition of carbon from the catalytic reaction. Carbon deposition is typically observed during the reaction of carbon dioxide or carbon monoxide with molecular hydrogen at iron oxide [53]. Interestingly, carbon can be (partially) removed from the surface by hydrogen, as the hydrogenation after run 6 indicates.

**Table 1:** Iron, oxygen, nitrogen, carbon and chloride content at the catalyst surface, as determined by XPS, as a function of catalytic runs.

sample	Fe	O	N	C	Cl

fresh catalyst	3.4	30	2.5	60.8	3.3
5 runs	11.8	38.5	1.9	46.7	1.2
10 runs	3.2	12	1.6	82.8	0.4
6 runs, followed by H_2_ reduction	12.9	43.8	1.3	41.2	0.8

**Figure 6:**
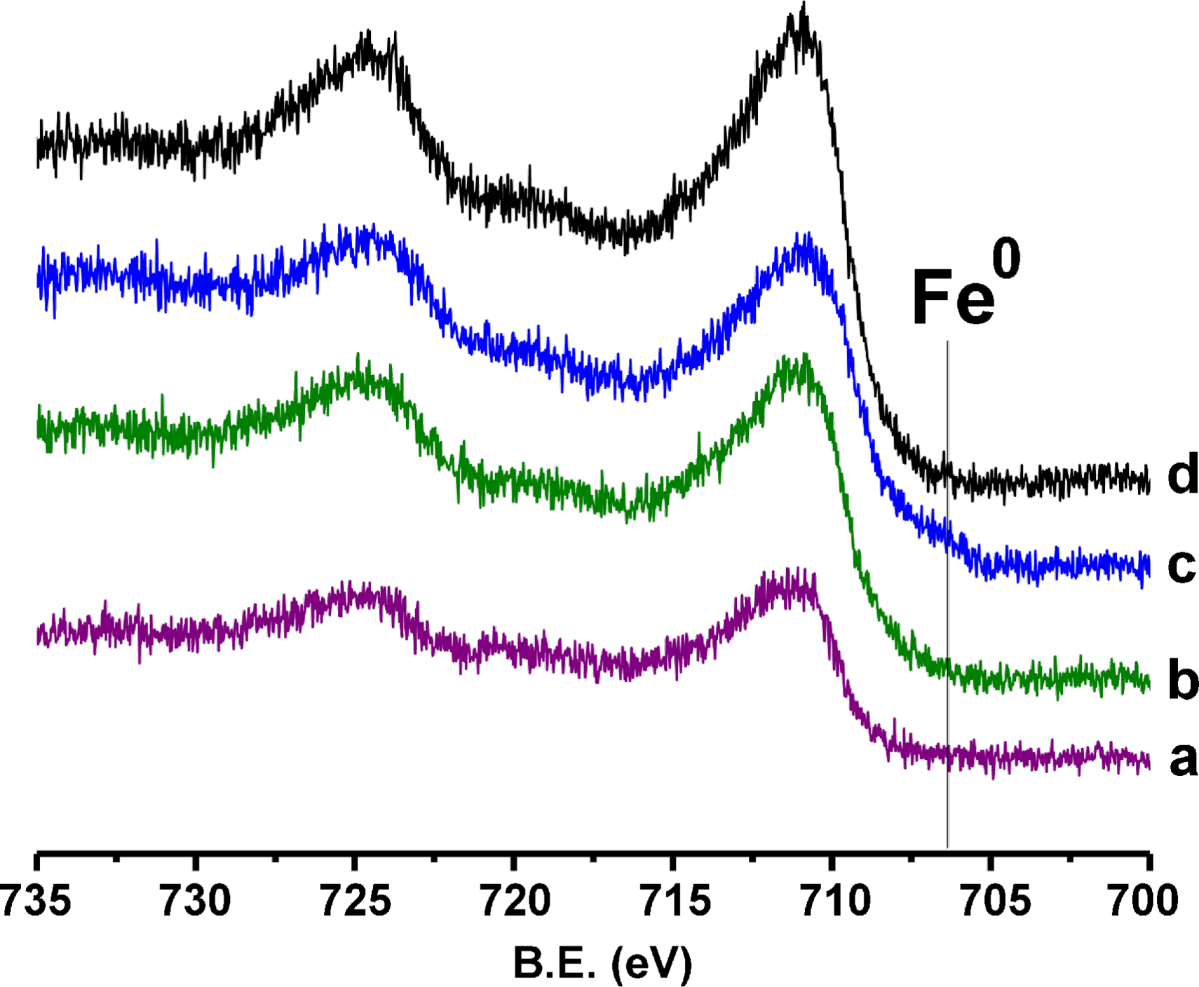
XPS of the Fe 2p^3/2^ and Fe 2p^1/2^ region for the catalyst: a) after 10 runs, b) after 5 runs, c) as prepared with HDA, d) after 6 runs, followed by H_2_ reduction.

The Fe 2p region (Figure 6) yields information about the oxidation state of iron in the particles. The small peak around 706.5 eV, which indicates Fe(0) is present in the fresh catalyst, but is absent after the catalyst has undergone 5 runs. It appears again in the sample that was reduced by H_2_. This indicates that the surface layer of the as-synthesized particles contains a small amount of Fe^0^ in addition to the Fe^2+^ and Fe^3+^, which is in agreement with the literature [54]. However, deconvolution of the Fe 2p region is rather difficult due to the large number of peaks necessary for an accurate curve fitting. From the experimental finding that the catalytic rate of the reaction remains practically unchanged at 0.00113 ± 0.00005 s^−1^g^−1^ for the consumption of CO_2_ at 400 °C, we have concluded that the presence of Fe(0) is not necessary for the observed formation of aromatic hydrocarbons. This multistep reaction (see below) proceeds apparently at the surface of freshly formed Fe_3_O_4_, which is produced through the reaction of H_2_O with Fe(0) in the temperature range in which the formation of aromatic hydrocarbons is observed [55].

#### Reaction mechanism

It is well accepted that the formation of aliphatic hydrocarbons from iron catalyzed CO_2_ hydrogenation reactions proceeds through a 2-step reaction process. In the first step, there is conversion of CO_2_ to CO via the reverse water-gas shift reaction (RWGS) [56].


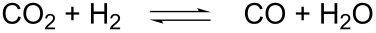


In the second step, hydrocarbon chains are built up through the Fischer–Tropsch reaction (FT) [57].


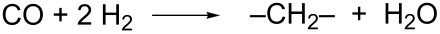


However, only methane (minor fraction), propane, butane and propanal could be identified as aliphatic products of the reaction of carbon dioxide and hydrogen at Fe/Fe_3_O_4_ nanocatalysts. Based on these findings and the characterization of the surfaces of the catalyst, we propose the following mechanism for the selective formation of aromatic hydrocarbons in the Fe/Fe_3_O_4_ nanoparticle catalyzed CO_2_ hydrogenation reaction. In the first step, the iron nanoparticle catalyzes the reverse water gas shift (RWGS) reaction to produce CO from CO_2_, as discussed above [56]. This step is the same as in the Fischer–Tropsch reaction [57]. In the second step of the manifold of reactions leading to aromatic products, the reaction of two CO molecules results in deposition of carbon on the surface of the catalyst and the formation of CO_2_. This “Boudouard reaction” is well established [53]:


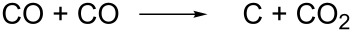


In the third step, two CO molecules react stepwise with the freshly deposited carbon on the catalyst to yield carbon suboxide (C_3_O_2_).


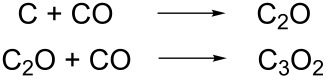


**Scheme 1:**
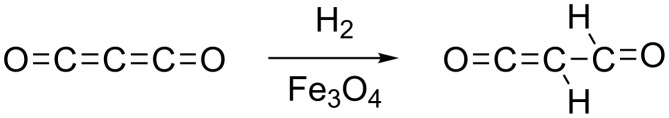
Hydrogenation of carbon suboxide.

This exothermic reaction has been reported in 1973 by Kammula and Shevlin [58]: C_3_O_2_ has been identified by comparison of the mass spectrum (Supporting Information File 1) with the reference spectrum available from the Wiley collection [59]. C_3_O_2_ is metastable and undergoes rapid polymerization at temperatures above 400 °C [60]. It is noteworthy that the formation of polymers at the surface of the catalyst was not observed in the reaction system studied here. Instead, C_3_O_2_ is reduced by H_2_ to H_2_C_3_O_2_ (Scheme 1, see mass spectrum in Supporting Information File 1).

**Scheme 2:**
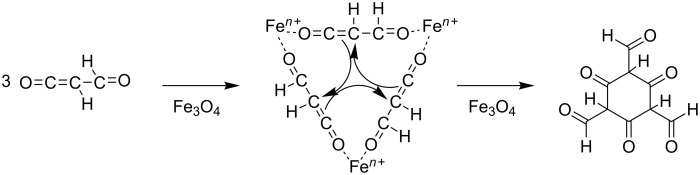
Trimerization of hydrogenated carbon suboxide on Fe_3_O_4_.

In the fourth step, H_2_C_3_O_2_ trimerizes at the surface of Fe_3_O_4_ to the symmetrical 2,4,6-trioxocyclohexane-1,3,5-tricarbaldehyde (Scheme 2).

Fe_3_O_4_ predominantly grows along the (111) and (001) planes, while (110) planes are less abundant [61]. The (001) plane can be viewed as alternating sequence of one layer with tetrahedrally coordinated Fe cations and a second layer with octahedrally coordinated Fe cations. Both coordination environments of Fe cations have been found at the surface [62–64]. A recent computational study found six possible terminations of the (111) plane. Again, two of them were found to be most stable, featuring either tetrahedrally or octahedrally coordinated Fe cations at the surface. Experimental evidence supporting these predictions is also available [65]. Further HRTEM experiments will have to be conducted to elucidate the exact nature of the active plane in Fe_3_O_4_, which is constantly formed from Fe and H_2_O under the working conditions of the CO_2_/H_2_ reaction to aromatic hydrocarbons [23].

**Scheme 3:**
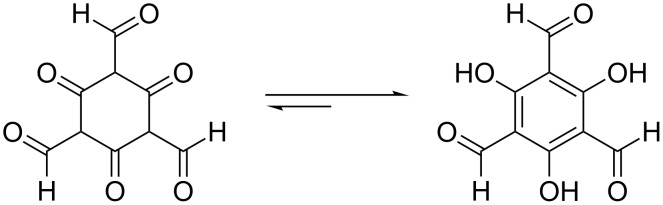
Keto–enol tautomerism leads to aromatization.

The fifth step consists of the keto–enol tautomerization from 2,4,6-trioxocyclohexane-1,3,5-tricarbaldehyde to 2,4,6-trihydroxybenzene-1,3,5-tricarbaldehyde (Scheme 3). This step is exergonic because of the aromatic resonance energy of the formed triphenol, which is approx. 30–35 kJ·mol^−1^ [66].

**Scheme 4:**
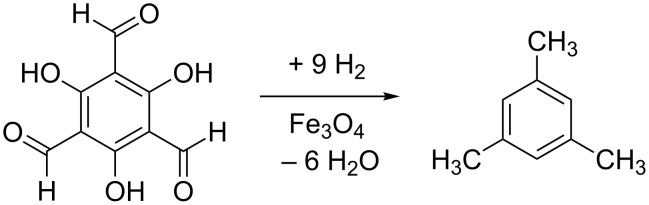
Reduction of the intermediate phenol derivative to mesitylene.

The symmetrical trihydroxybenzene-derivative is then reduced to the principal reaction product mesitylene (C_9_H_12_) (Scheme 4).

**Scheme 5:**
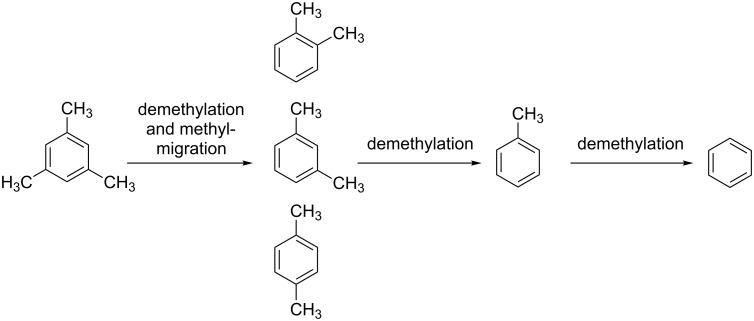
Demethylation is at this stage of mechanistic research the most likely process explaining the formation of xylenes, toluene, and benzene. Further explanations are provided in the text.

At high temperature in the presence at Fe_3_O_4_, mesitylene undergoes stepwise demethylation to form xylenes, toluene and finally, benzene (Scheme 5). The byproduct of this reaction consists of a mixture of aliphatic hydrocarbons. Note that the reduction of C_3_O_2_ by 6 H_2_ is also able to form propane, which, therefore, does not necessarily have to come from demethylation processes.

It is noteworthy that all aromatic reaction products (mesitylene, *o*-, *m*-, and *p*-xylene, toluene and benzene) are high-octane components of gasoline [67]. Therefore, this catalytic reaction is able to transform gaseous hydrogen and carbon dioxide into high-quality fuel, which can be distributed by using the existing distribution infrastructure. Our findings are corroborated by Wright et al. (US patent 4,565,831), in which the authors describe a process for producing a mixture of aliphatic and aromatic hydrocarbons from carbon monoxide and water at modest temperatures on iron/thallium catalysts [68]. Although no mechanisms are discussed, it is of importance that Wright et al. also found that aromatic hydrocarbons are easily formed on Fe_3_O_4_. The latter is formed from Fe in the presence of H_2_O in the temperature range of interest.

Finally, it should be noted that carbon suboxide undergoes thermolysis to carbon monoxide and dicarbon monoxide (C_2_O) [69]. Although this process is observed at distinctly higher temperatures in the gas phase, it may also occur at the surface of the catalyst. It is very likely that dicarbon monoxide will then be hydrogenated to ketene (H_2_C=C=O). The presence of ketene would offer a pathway to xylenes, toluene, and benzene without the need to postulate a demethylation mechanism of mesitylene.

#### Tests of the mechanistic paradigm

To verify the formation of mesitylene, the CO_2_ hydrogenation reaction was carried out at 520 °C using exactly the same conditions as described previously. A peak at 10.417 min, corresponding to *m*/*z* 120 was observed in the GC–MS. This compound was identified as mesitylene by comparing with a standard sample. This proved unambiguously that mesitylene is formed from CO_2_ and H_2_ at the surface of the Fe/Fe_3_O_4_ nanocatalysts.

#### Product selectivities

**Figure 7:**
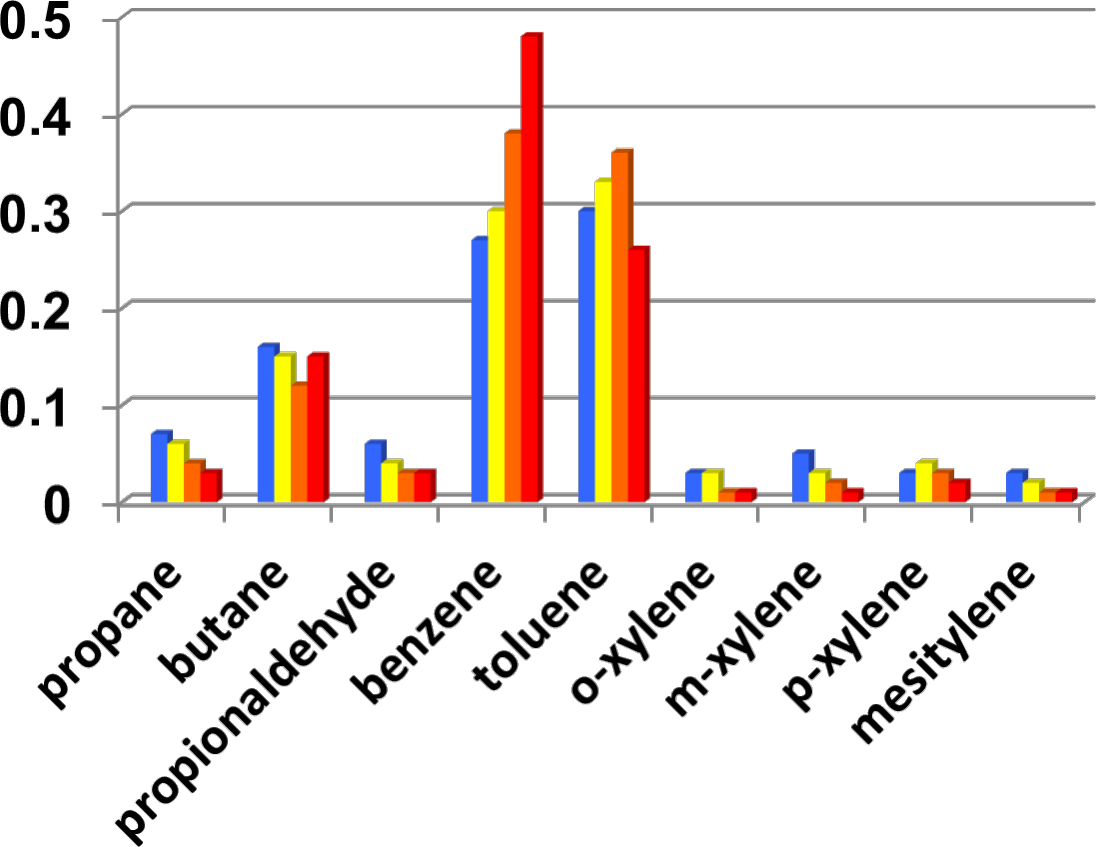
Product selectivities as a function of temperature: blue: 440 °C, yellow: 480 °C, orange: 500 °C, red: 520 °C. The experimental error from three repetitions is +/−0.03 for butane, benzene and toluene, and 0.01 for all other identified reaction products.

We have calculated the product selectivities, defined as the number of moles of a specific product over the total number of moles of product, based on the GC–MS results obtained at 440 °C, 480 °C, 500 °C, and 520 °C and our calibration data using chemical standards. The results obtained for all reaction products that were clearly identified are summarized in Figure 7.

## Experimental

### Synthesis of the Fe/Fe_3_O_4_ nanocatalysts

Iron nanoparticles were prepared with slight modification of a literature procedure described by Lacroix et al [49]. A 250 mL, three-necked, round-bottom flask equipped with a magnetic stir bar, one cold water cooled jacketed condenser on the middle neck, one septum and one temperature probe on each of the outer necks is charged with 60 mL 1-octadecene (ODE), 0.9 mL oleylamine and 0.831 g hexadecylammonium hydrochloride (HDA·HCl). The reaction system was connected to a Schlenk line through the top of the jacketed condenser. The reaction mixture was degassed at 120 °C for 30 min under vigorous stirring. After being refilled with argon, the reaction mixture was heated to 180 °C. Three portions of 0.7 mL Fe(CO)_5_ were injected into the reaction mixture via a syringe every 20 min. The reaction mixture was kept at 180 °C for another 40 min after the last injection, and allowed to cool to room temperature. The supernatant was decanted, and the iron nanoparticles accumulated on the magnetic stir bar were washed with hexane (5 × 10 mL) and ethanol (5 × 10 mL). The product was then dried in vacuum. Based on iron, the yield of the reaction was 95%.

#### Catalytic reduction of CO_2_/H_2_ mixtures to aromatic hydrocarbons

The CO_2_/H_2_ mixture (1:1 mol/mol) was heated for 4 h in a tubular reactor (*d* = 20 mm) containing 50 mg of iron nanocatalyst in a porcelain microvessel in a quartz reaction tube (inner diameter = 0.80 cm, *l* = 40 cm). After the reaction mixture was allowed to cool down to rt, the gas phase was analyzed by using GC–MS. The reaction temperature was between 380 °C and 520 °C.

**Figure 8:**
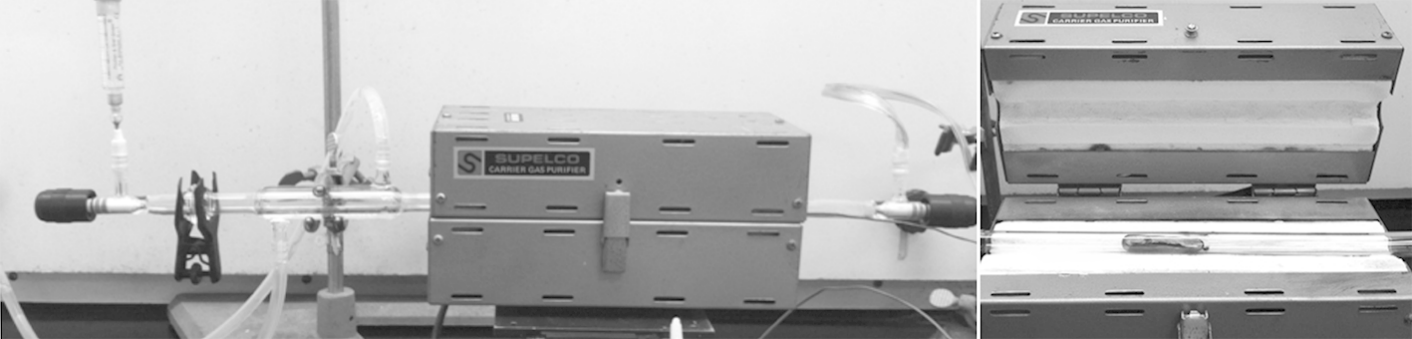
Tubular reactor used in the catalytic reduction reaction.

GC–MS analysis was carried out on an Agilent Technologies 7890A GC/5975C VL MSD with triple-axis detector using a HP5 capillary column. The compounds were identified by comparing their MS spectra with the standards in the Wiley data base, as well as by co-injection.

Powder X-ray diffraction (XRD) patterns were obtained on a Bruker D8 X-ray diffractometer with Cu Kα radiation.

**Transmission electron microscopy (TEM):** Sample preparation and data collection are similarly described in a previous paper [70]. Briefly, samples were prepared by suspending the catalyst in ethanol and agitating in an ultrasonic bath for 15 min. A catalyst sample (10 µL) was placed onto copper mesh grid with lacey carbon film. The wet grids were allowed to air-dry for several minutes prior to being examined under TEM. The catalyst particle size and morphology were examined by bright-field and dark-field transmission electron microscopy (TEM) using an FEI Technai G_2_ transmission electron microscope at an electron acceleration voltage of 200 kV. High resolution images were captured using a standardized, normative electron dose and a constant defocus value from the carbon-coated surfaces.

**X-ray photoelectron spectroscopy (XPS):** Sample preparation and data collection are similarly described in a previous paper [71]. Data was recorded with a Perkin-Elmer PHI 5400 electron spectrometer using acrochromatic Al Kα radiation (1486.6 eV). Analysis was carried out under vacuum (*p* < 5·10^−9^ Torr) and heated to 120 °C to remove any adsorbed molecules on the surface. The XPS binding energies were measured with a precision of 0.025 eV. The analyzer pass energy was set to 17.9 eV, the contact time was 50 ms, and the area scanned was 4 mm^2^.

## Conclusion

We have obtained evidence for the formation of aromatic hydrocarbons (benzene, toluene, xylenes and mesitylene) from carbon dioxide and hydrogen mixtures at 1 atm on Fe/Fe_3_O_4_ nanocatalysts. A minor fraction of aliphatic hydrocarbons is formed as well. This finding offers a viable pathway towards the direct and efficient formation of hydrocarbon mixtures that are suitable as chemical starting materials and high-quality biofuels from CO_2_ and hydrogen. This technology is, principally compatible with solar heat and hydrogen technology and has the potential to mitigate the impacts of global warming by making use of the existing distribution technology for gasoline.

## Supporting Information

CO_2_ conversion data at 400 °C, characterization of all relevant intermediates and products by GC–MS, and XRD analysis of the catalyst before and after catalysis are supplied as Supporting Information.

File 1 Additional experimental data.
