# Supplementary material for: Carbon dioxide hydrogenation to aromatic hydrocarbons by using an iron/iron oxide nanocatalyst
Source: Beilstein J Nanotechnol. 2014 Jun 2;5:760–9. doi: 10.3762/bjnano.5.88 (PMC4077464; doi:10.3762/bjnano.5.88)
Supplement: File 1 — Additional experimental data. [file Beilstein_J_Nanotechnol-05-760-s001.pdf]

Supporting Information

for

Carbon dioxide hydrogenation to aromatic hydrocarbons by using an iron/iron oxide nanocatalyst

Hongwang Wang*[1], Jim Hodgson[1], Tej B. Shrestha[2], Prem S. Thapa[3], David Moore[3], Xiaorong Wu[4], Myles Ikenberry[5], Deryl L. Troyer[2], Donghai Wang[4], Keith L. Hohn[5] and Stefan H. Bossmann*[1]

Address: [1]Kansas State University, Department of Chemistry, 201CBC Building, Manhattan, KS 66506, USA, 001-785-532-6817; [2]Kansas State University, Department of Anatomy & Physiology, 130 Coles Hall, Manhattan, KS 66506, USA; [3]University of Kansas, Microscopy and Analytical Imaging Laboratory, 1043 Haworth, Lawrence, KS 66045, USA; [4]Kansas State University, Department of Biological and Agricultural Engineering, 150 Seaton Hall, Manhattan, KS 66506, USA and [5]Kansas State University, Department of Chemical Engineering, 1016 Durland Hall, Manhattan, KS 66506, USA

Email: Hongwang Wang* - hongwang@ksu.edu; Stefan H. Bossmann* - sbossman@ksu.edu

*Corresponding author

Additional experimental data

Reaction kinetics

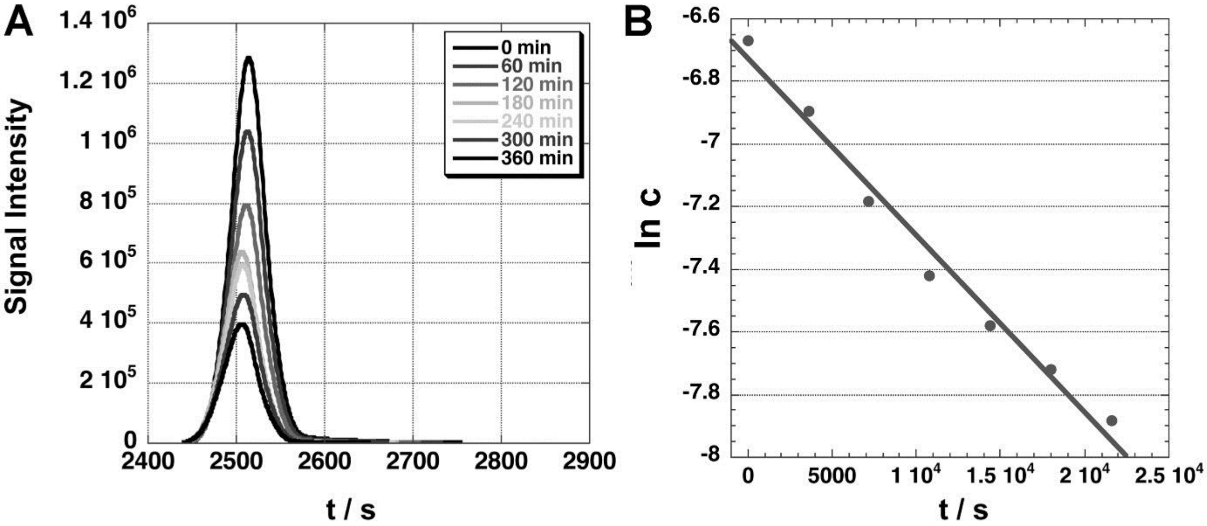

Figure S1: Consumption of CO_2 at 400 °C. A: CO_2 peak as a function of reaction time, as recorded by GC–MS. B: The reaction is a heterogeneous first order kinetics, as the plot ln(signal intensity) vs t indicates.

Additional GC–MS chromatograms and mass spectra

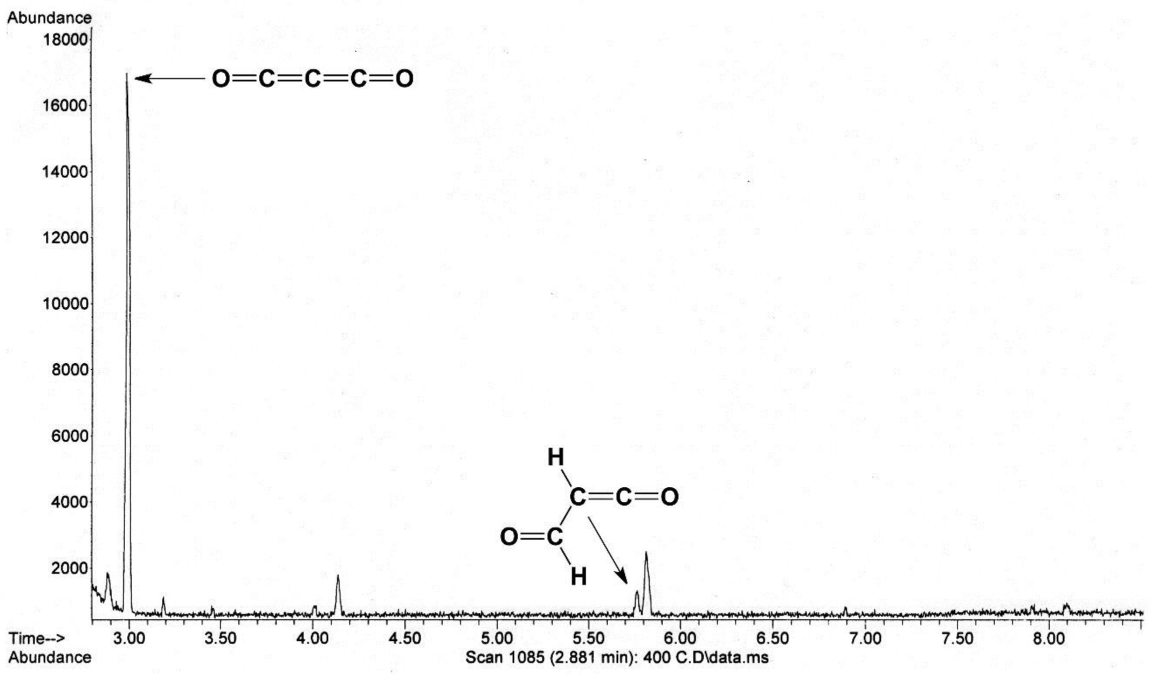

Figure S2: GC–MS chromatogram showing the major reaction intermediates that are formed at 400 °C.

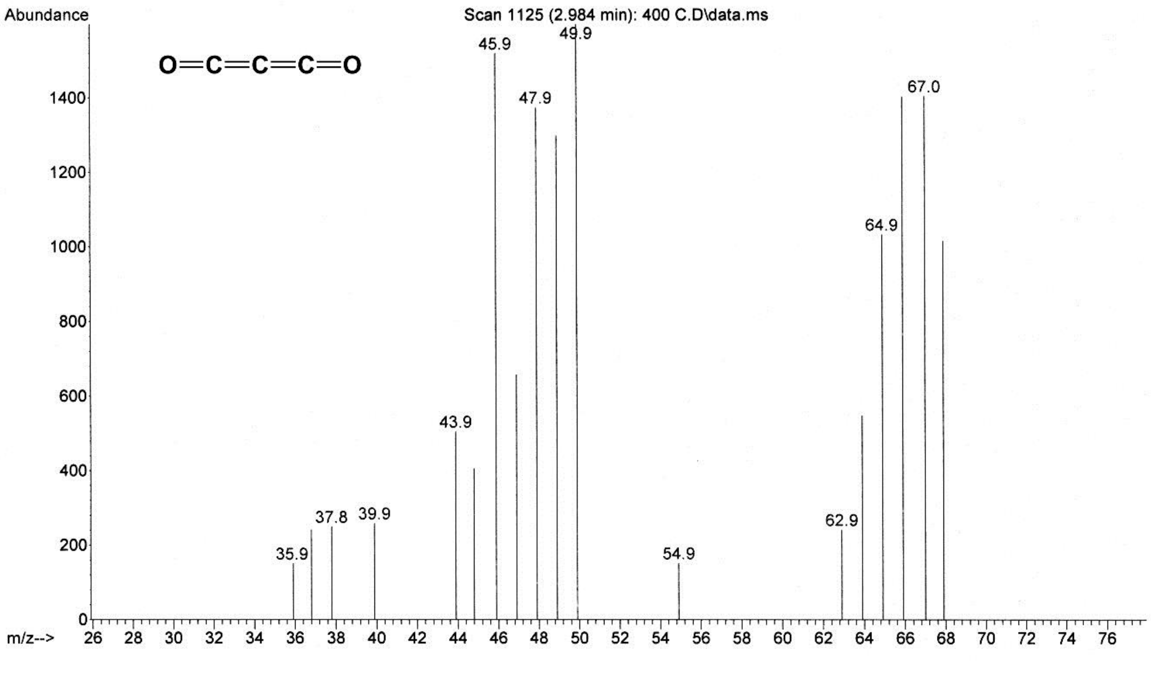

Figure S3: Mass spectrum of carbon suboxide (C_3O_2).

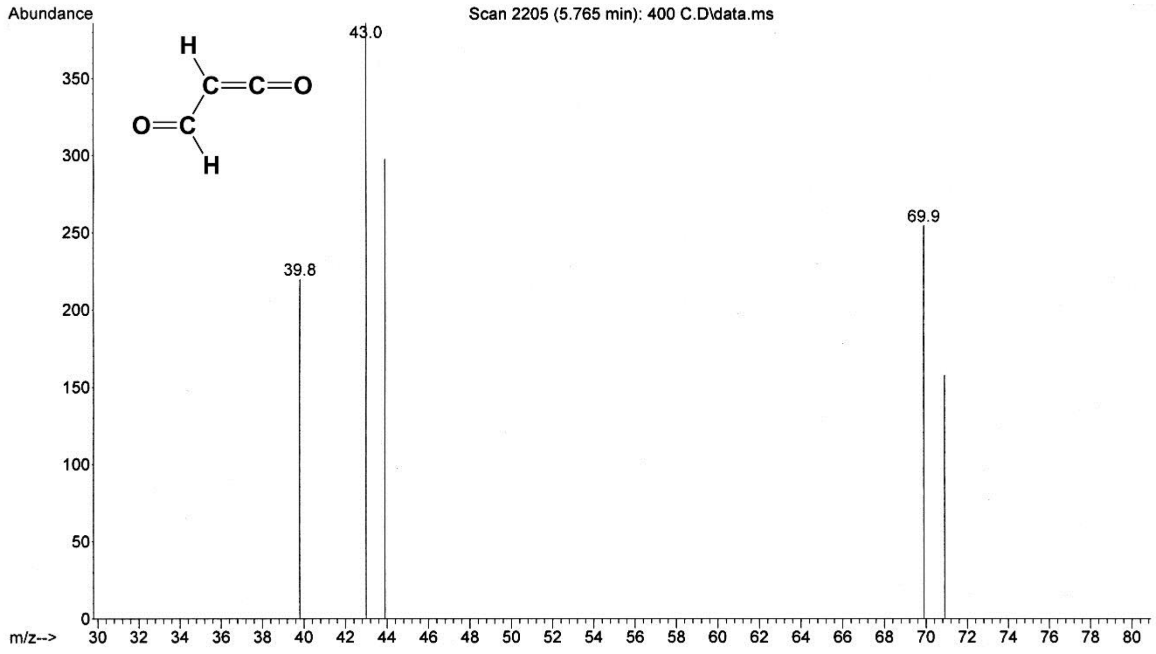

Figure S4: Mass spectrum of 3-oxoacrylaldehyde ($C_3H_2O_2$).

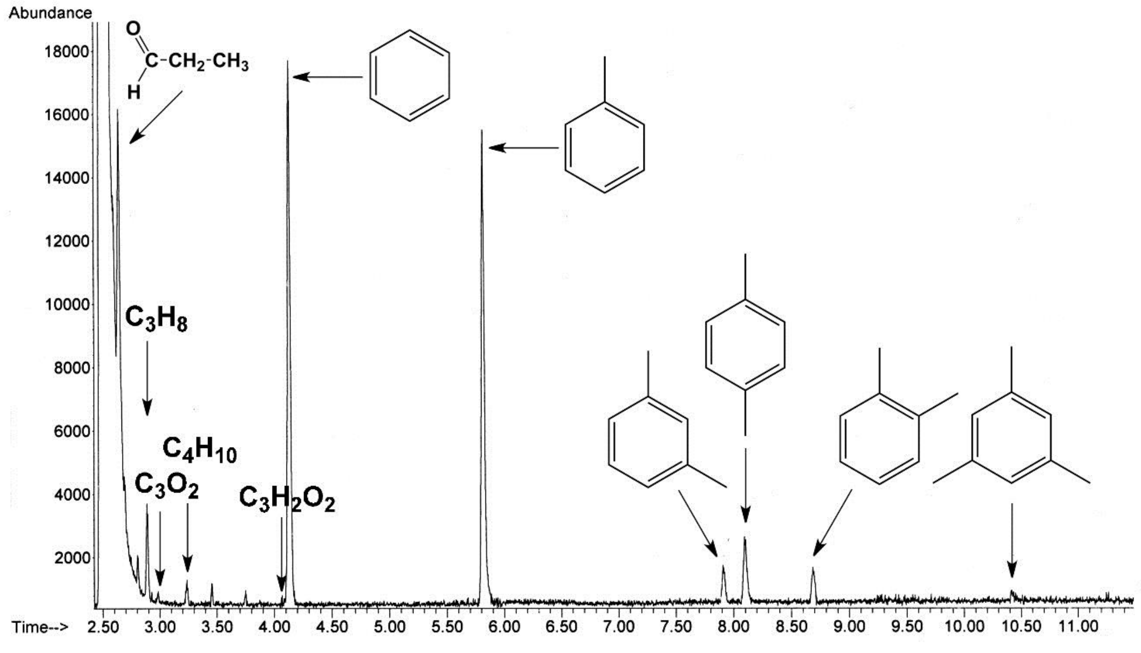

Figure S5: GC–MS chromatogram showing the major reaction products that are formed at 500 °C.

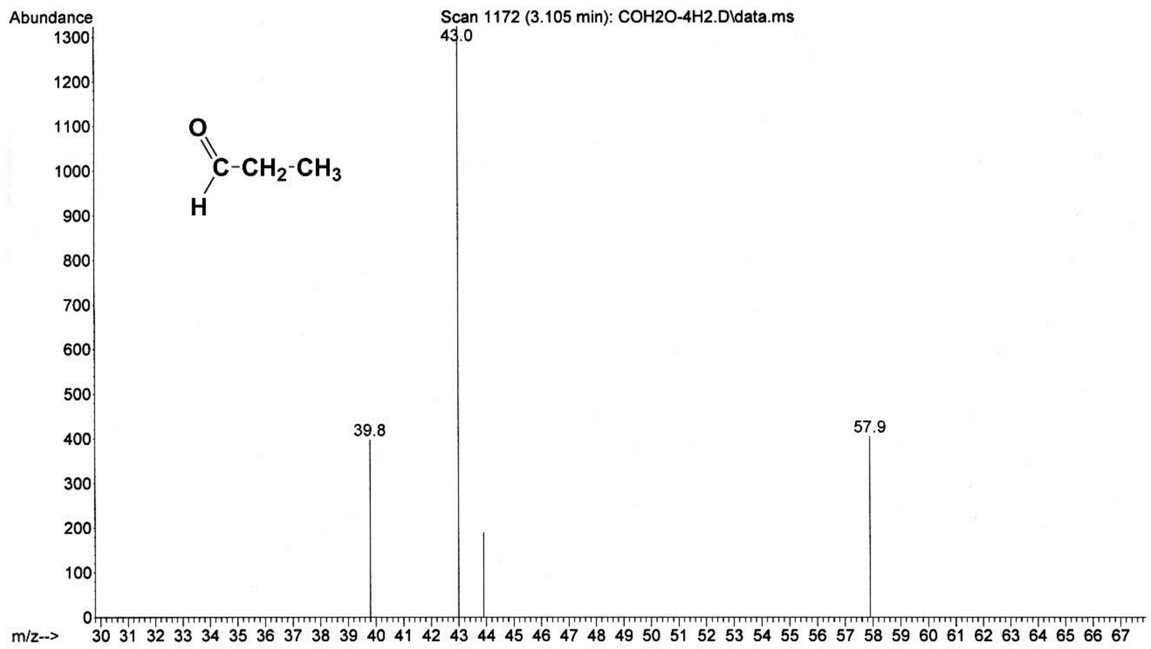

Figure S6: Mass spectrum of propionaldehyde (C_3H_5O).

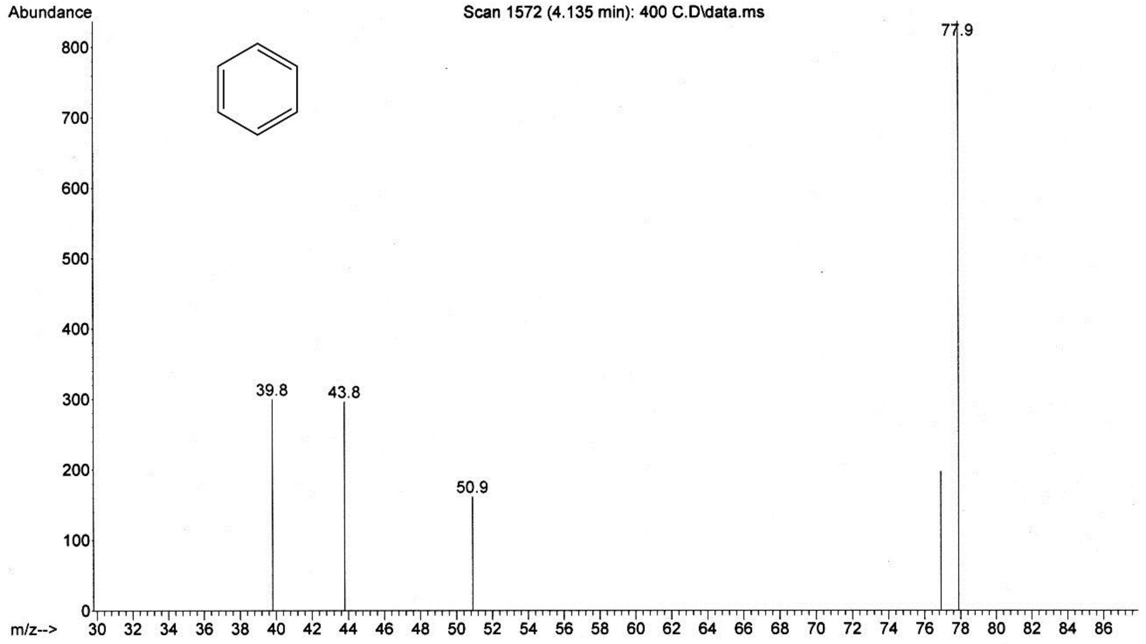

Figure S7: Mass spectrum of benzene (C_6H_6).

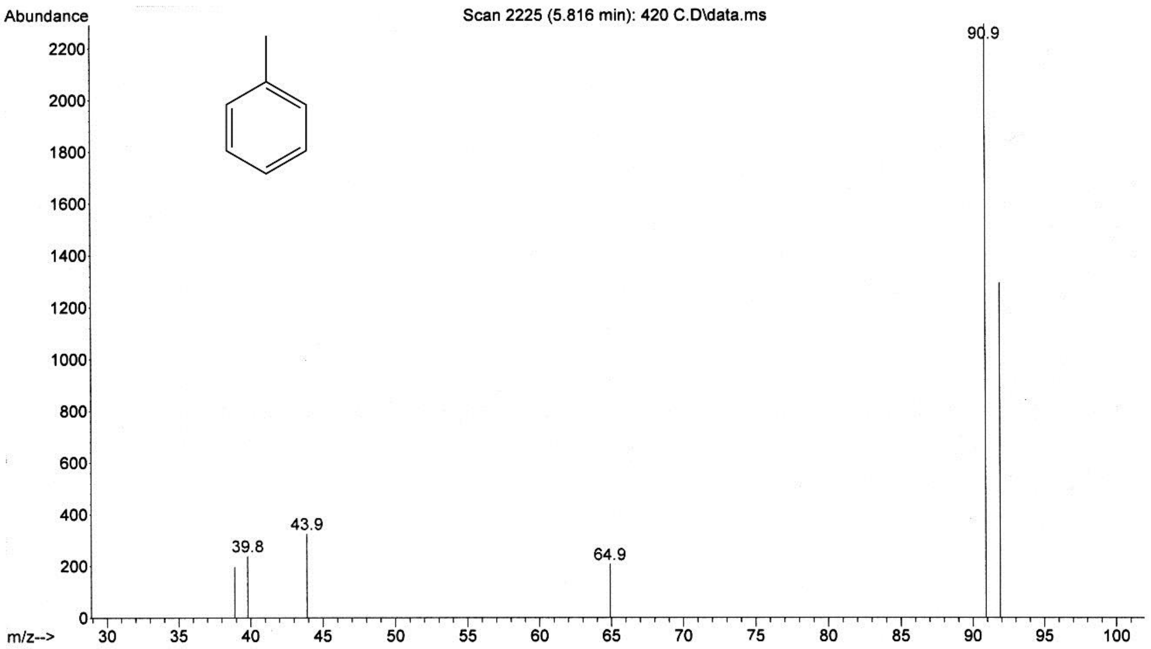

Figure S8: Mass spectrum of toluene (C_7H_8).

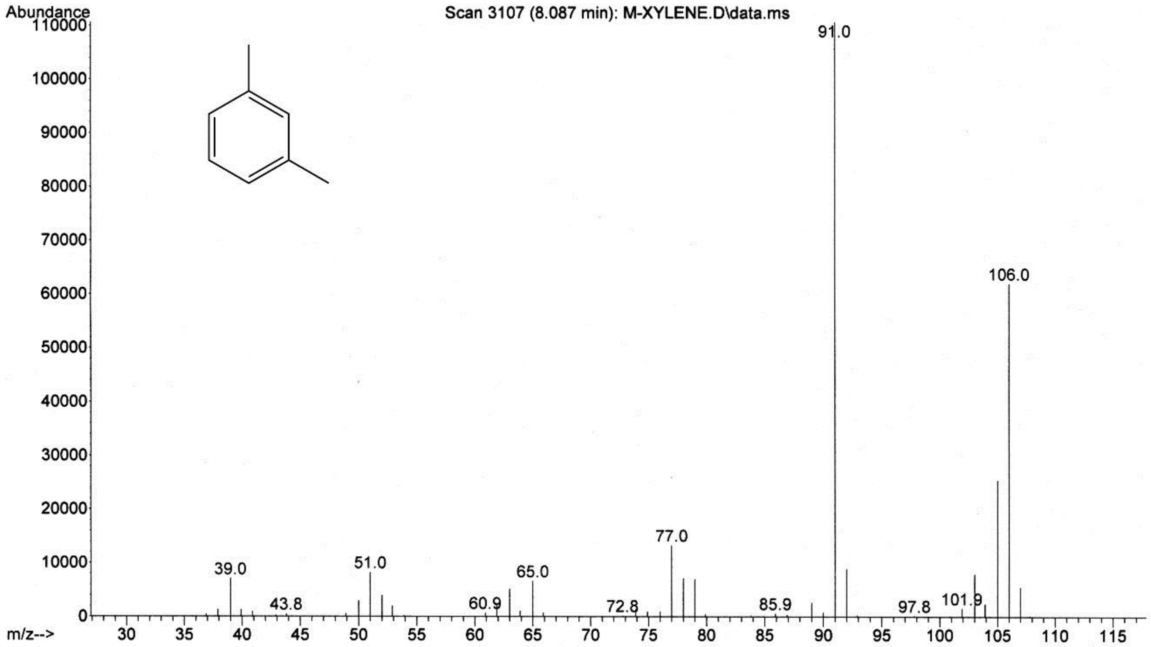

Figure S9: Mass spectrum of *meta*-xylene (C_8H_{10}).

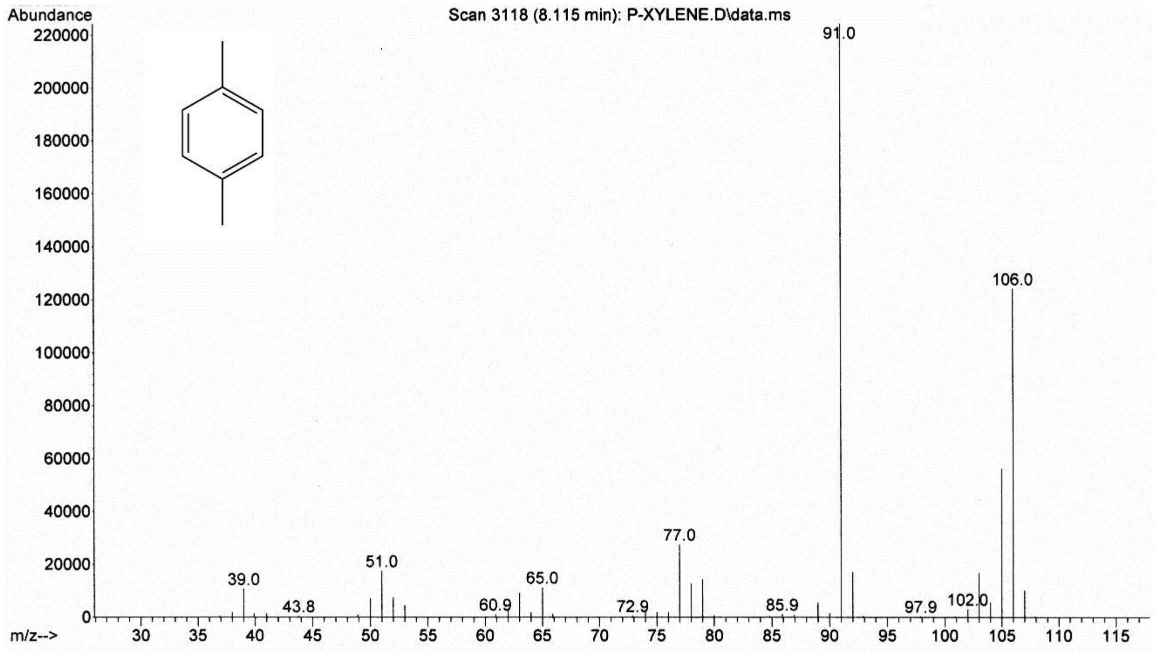

Figure S10: Mass spectrum of *para*-xylene (C_8H_{10}).

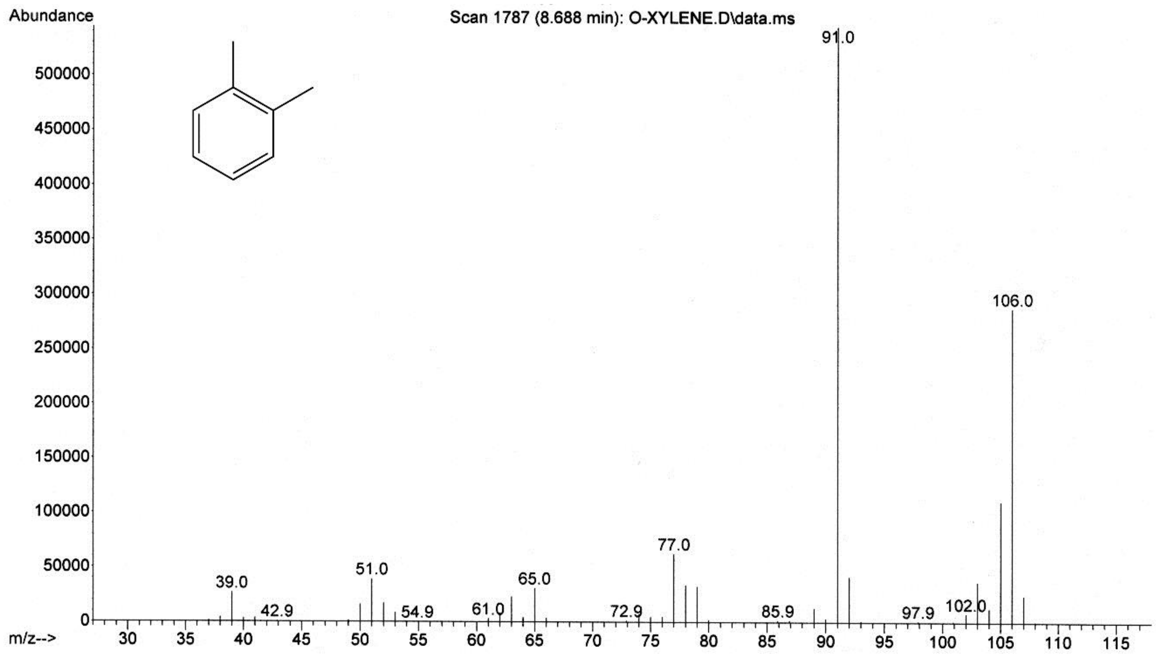

Figure S11: Mass spectrum of *ortho*-xylene (C_8H_{10}).

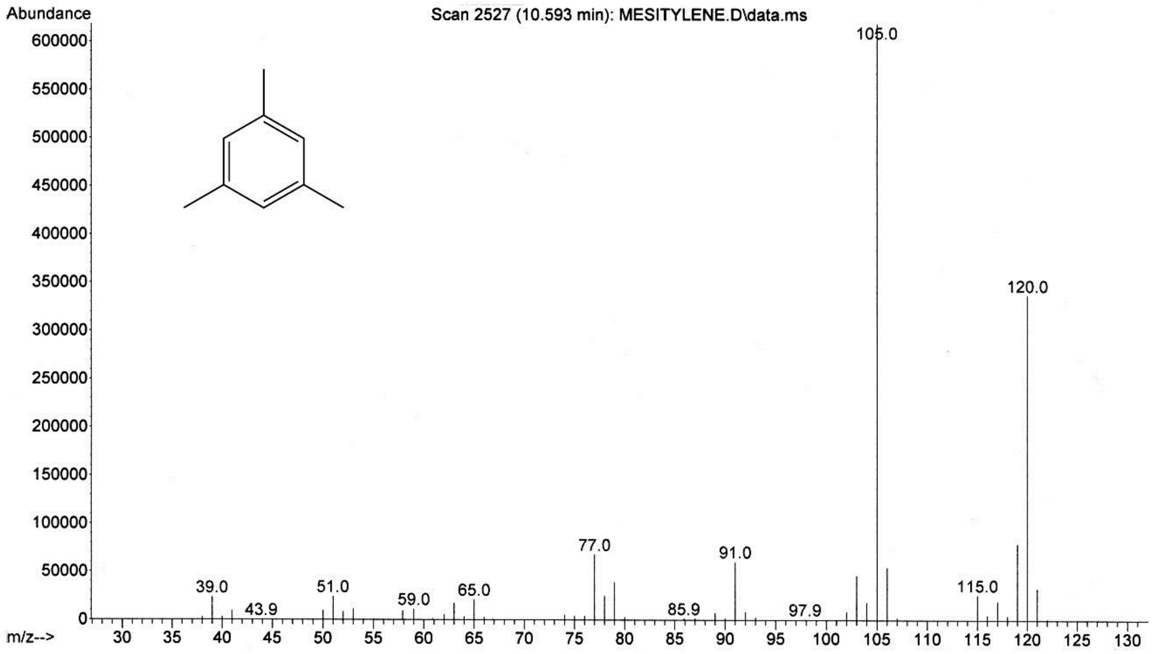

Figure S12: Mass spectrum of mesitylene (C_9H_{12}).

Additional XRD Spectra

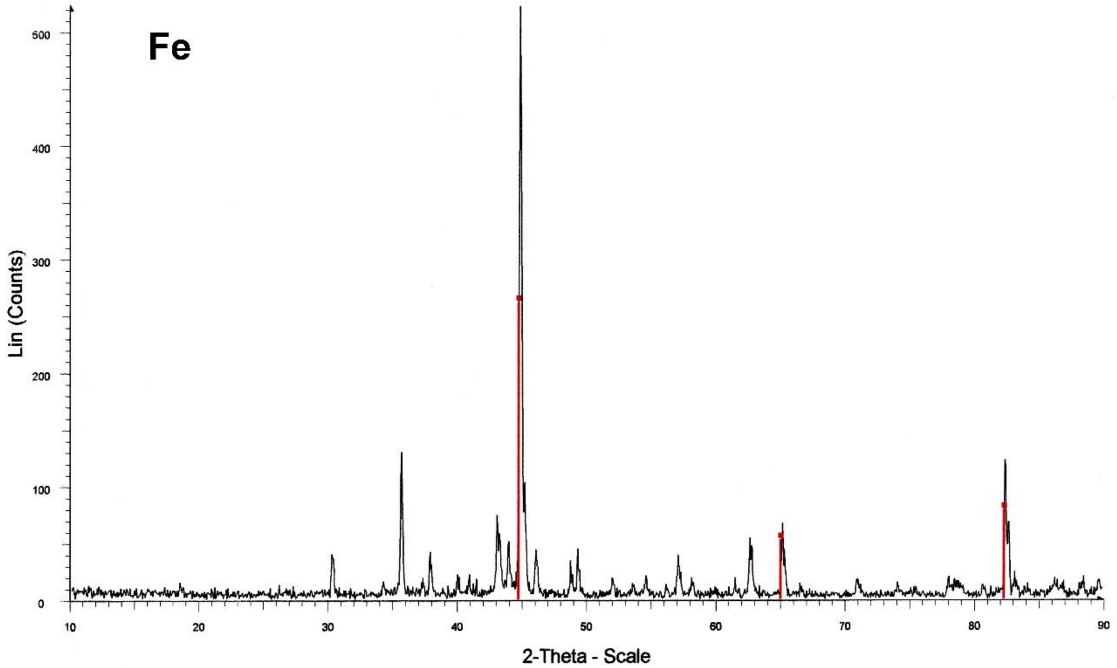

Figure S13: X-ray diffractogram of the Fe/Fe_3O_4-catalyst after 5 catalytic runs (2 h each).

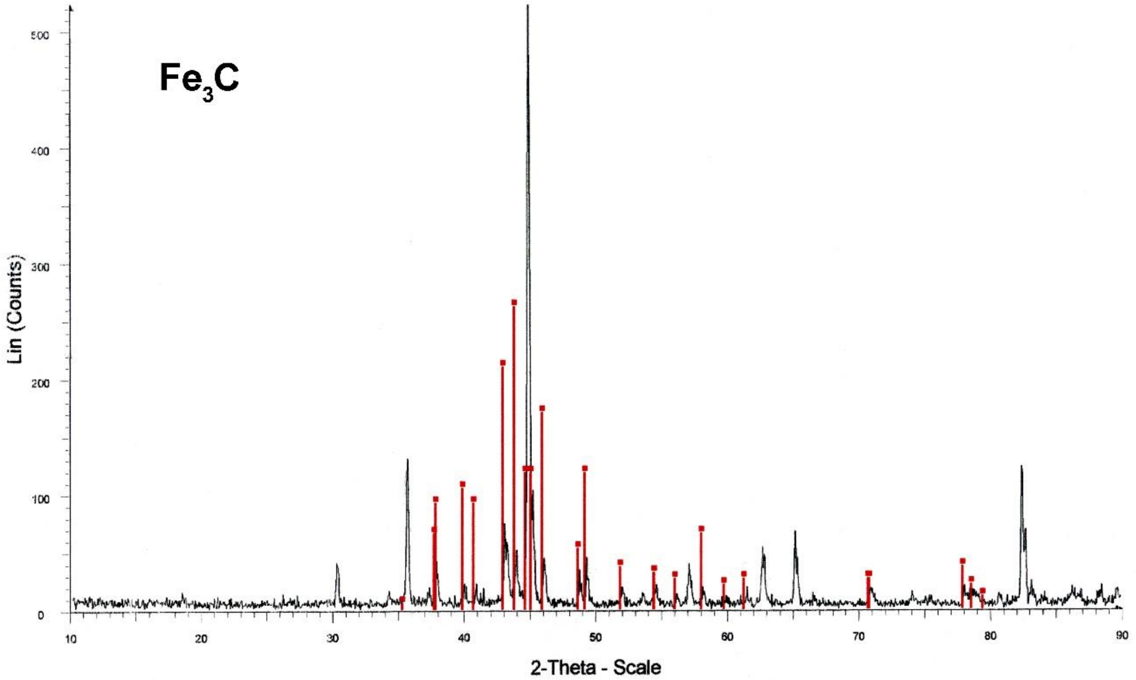

Figure S14: X-ray diffractogram of the Fe/Fe_3O_4-catalyst after 5 catalytic runs (2 h each).

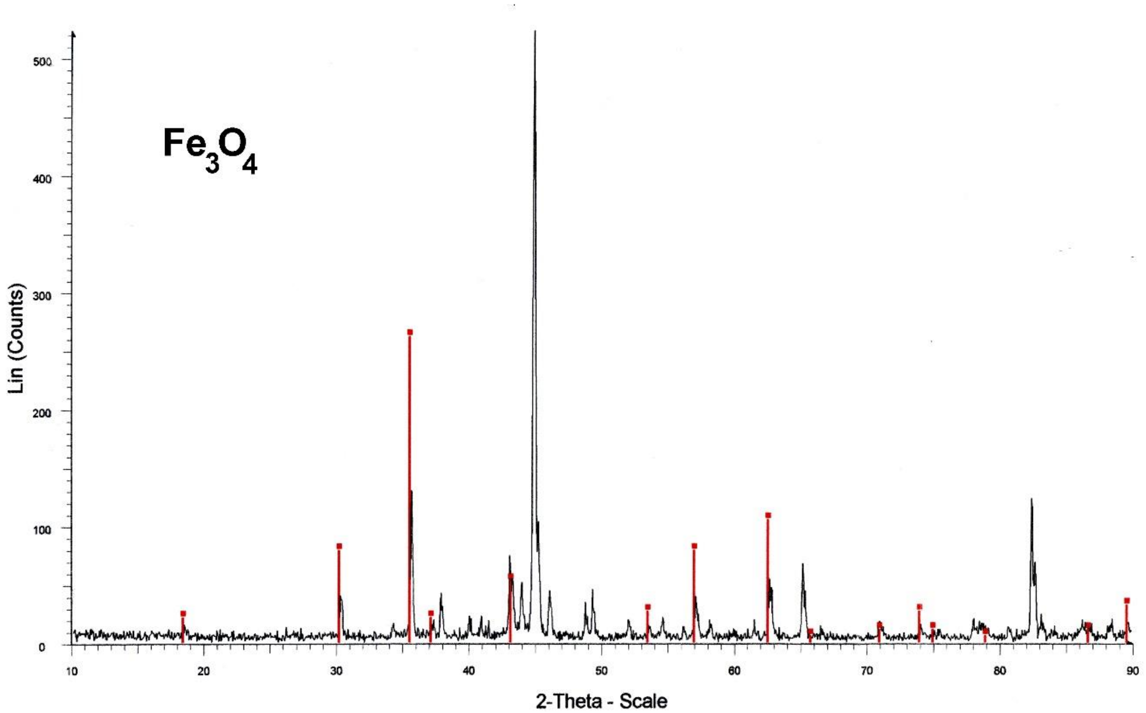

Figure S15: X-ray diffractogram of the Fe/Fe_3O_4-catalyst after 5 catalytic runs (2 h each).
